# Community health workers and promotoras’ perspectives of a research best practice course: A focus group study

**DOI:** 10.1017/cts.2022.464

**Published:** 2022-09-26

**Authors:** Deepthi S. Varma, Elias Samuels, Gretchen Piatt, Daphne C. Watkins, Meghan Spiroff, Linda B. Cottler, Sergio Aguilar Gaxiola, Susan L. Murphy

**Affiliations:** 1 Department of Epidemiology, College of Public Health and Health Professions and College of Medicine, University of Florida, Gainesville, FL, USA; 2 Michigan Institute of Clinical and Health Research, University of Michigan, Ann Arbor, MI, USA; 3 Department of Learning Health Sciences, Department of Health Behavior and Health Education, School of Public Health, University of Michigan, Ann Arbor, MI, USA; 4 School of Social Work, Vivian A. and James L. Curtis Center for Health Equity Research and Training, University of Michigan, Ann Arbor, MI, USA; 5 Department of Obstetrics and Gynecology, University of Michigan, Ann Arbor, MI, USA; 6 Department of Internal Medicine, University of California, Davis, CA, USA; 7 Department of Physical Medicine and Rehabilitation, University of Michigan, Ann Arbor, MI, USA

**Keywords:** Community health workers, promotoras, community engagement, research ethics, training

## Abstract

**Introduction:**

Community Health Workers and Promotoras (CHW/Ps) are valued for their role in helping to engage community members in research. CHW/Ps have traditionally received variable training in research fundamentals, including importance and promotion of research rigor to establish consistency in the methods used over time. Research best practices training exists for research professionals, but no standard training is provided as part of the CHW/P job role. To develop this CHW/P research best practices training, our team engaged English- and Spanish-speaking CHW/Ps to watch an early version of an online module and to examine perceptions of the relevance of such a training and optimal delivery methods.

**Methods:**

Six virtual focus group discussions were conducted (three in English and three in Spanish) across different US geographic regions with currently employed CHW/Ps.

**Results:**

Forty CHW/Ps participated (95% female, mean age 44 years, 58% identifying as Hispanic/Latino). Four themes emerged: relevance of training, benefits of providing a certificate of completion, flexible training delivery modalities, and peer-led training.

**Discussion:**

With participation from representatives of the intended learner group of CHW/Ps, our team found that CHW/Ps valued learning about research best practices. They perceived culturally- and linguistically appropriate health research training to be highly relevant to their role, particularly for communicating key information to community members about their participation in health research. Additionally, participants provided input on effective dissemination of the training including the benefit of having proof of course completion, involvement of peer trainers, and value of providing the option to participate in online training.

## Introduction

Community Health Workers and Promotoras (CHW/Ps) are gaining recognition as critical partners in community-engaged research [1–3]. These professionals constitute an important component of the health research workforce and are distinctive for being credible and trusted members of communities with which they often share culture, socioeconomic status, lived experience, and language. By virtue of this experience and expertise, CHW/Ps have an almost unrivaled capacity to understand and represent the health and research priorities of the communities they serve. CHW/Ps are employed as team members in research studies, often serving as the effective bridge between researchers and communities. Thus, they are uniquely suited to meaningfully engage community members in research, especially in communities that are underserved and/or distrustful of academic research institutions. CHW/Ps link community members to health-related research opportunities, promote health and research literacy, and help to empower community members to make informed choices about participating in research studies [4].

Despite the important role that CHW/Ps have in supporting community-engaged research, training in research best practices for CHW/Ps is variable [5,6], and there are challenges related to CHW/Ps’ understanding and application of research best practices [7–9], particularly in practice-related topics like participant enrollment and adverse event reporting. CHW/Ps who serve formally on research teams receive general human participants training, which may be ill-suited to their research role or to the unique context of underserved areas. In addition, many existing trainings have not been adapted to be culturally or linguistically appropriate for CHW/Ps serving those communities. Finally, delivery of trainings may not be offered in a manner that would optimally suit CHW/Ps and support consistency in implementation of research best practices within and across community sites. While available seminars and online training provided one time at the beginning of a project may offer some useful information for supporting research best practices for CHW/Ps, actions related to supporting research over time in communities are unlikely to be transferred to the workplace without reinforcement [10,11].

With the increasing recognition and utilization of CHW/Ps supporting research in communities, there is a critical need for research training appropriate for this growing workforce. Without sufficient opportunities for training, CHW/Ps may not be prepared to foster understanding among study teams and community members about what it means to implement the research study in which they are involved. Similar to good clinical practice for researchers, best practices training facilitates an understanding of the knowledge, actions, and behaviors needed to support research quality in communities, including but not limited to key aspects of human respondents’ protections [12]. This type of training could help CHW/Ps become more aware of issues that can arise in research conducted in their communities that could affect the rigor of the studies, such as potentially being coercive in recruiting people due to their being a trusted member of the community or the difficulty in maintaining confidentiality of participants when they are part of their extended social network [9,13,14]. The availability of this important training may also foster the integration of CHW/Ps into learning health systems that promise to improve the design, implementation, and impact of health research across the translational spectrum.

Academic research institutions funded by NIH for Clinical and Translational Science Awards (CTSAs) across the nation work together to enhance translation of research discovery in vital partnerships with strong community engagement programs [15,16]. Because these institutions often partner with CHW/Ps as part of their community-engaged research programs, this network can provide structure for research best practices training for CHW/Ps and help strengthen community–academic partnerships. In alignment with the principles of successful community-engaged health promotion and research, CTSAs should ensure stakeholder involvement at every step of the research process for any aspect of community engagement to be acceptable and effective among all [4].

Thus, a community-engaged research approach was used to identify key aspects of the training design and delivery for this project [4]. We invited CHW/Ps from diverse backgrounds and from three different geographical locations within the USA to gain insight into perspectives from CHW/Ps on how research best practices training can be efficiently and effectively disseminated throughout the CTSA Consortium and broadly nationwide. This approach enabled this team to identify how English and Spanish versions of research best practices training could be customized for distribution to practicing CHW/Ps working across the nation.

This paper presents findings from a series of focus groups conducted to understand the relevance of the newly developed training modules, benefits of training certificates for CHW/Promotoras, preferred delivery methods of CHW/ Promotoras training modules, and the barriers and facilitators to peer-led training. These focus groups were conducted to ensure that the training being developed was appropriate culturally and linguistically, that it can be effectively disseminated, and that it is impactful to CHW/Ps and to academic researchers who partner with them. This process was implemented with support from a U01 grant awarded to three CTSA-funded institutions by a team of health researchers with deep expertise in community engaged health research and Good Clinical Practice research training.

## Materials and Methods

We conducted six virtual focus group discussions, three in English and three in Spanish, with 40 CHWs and Promotoras (CHW/P) working at the three affiliated universities on the project. Each university had established partnerships with local and state community-based organizations that served as the basis of recruitment of CHW/Ps for the focus groups in each geographical region. The University of Michigan site conducted two English focus groups, the University of California at Davis had one Spanish focus group and the University of Florida had two focus groups (one in English and one in Spanish). The sixth focus group was conducted in Spanish across institutions; it was conducted with participants from Michigan and Florida, with a facilitator from the California team. This group was the last conducted as the results were subsequently determined to have achieved data saturation [17]. All six focus groups were conducted between February and July 2021.

### Recruitment

Participants were currently practicing CHWs/Ps who were contacted through Community Health Worker/Promotora coalitions, study flyers, emails to community-based organizations at each site, and through word of mouth. Potential participants completed a brief survey via a link in the email or flyer or contacted the study coordinator at the site to complete the demographic and screening questions by phone. The CHW/Ps were selected to include diverse ages, races, ethnicities, language, and years of CHW/P work experience. Participants were given a choice of dates and times or assigned to one by the coordinator; each participant received at least one reminder before the focus group was held.

### Data Collection

All focus groups were conducted virtually via Zoom by a trained facilitator using IRB-approved protocols and interview guides in English and Spanish. We followed World Health Organization (WHO) guidelines for translations and adaptations protocol to create the Spanish language focus group materials. The Spanish language focus groups were conducted by trained facilitators who were fluent in English and Spanish, along with a note-taker. All focus groups were conducted following a protocol that included the introduction, ground rules of interaction, focus group questions, and prompts. The semi-structured interview guide was developed based on the research objectives (Table 1). All participants completed the online registration form before participating. Participant data collected included age, gender, race, ethnicity, language, and years of work experience as a CHW/P. All focus groups were recorded, then translated, and transcribed.

**Table 1.** Interview guide topics & questions

Topic	Questions
Relevance of proposed training	What do you think about the relevance of these proposed topics for the training?
	What do you think is most important for Community Health Workers or Promotoras to know? Is there anything missing?
	Where can we make improvements?
Training delivery methods	What do you think about the different ways we are delivering this training?
Barriers & facilitators to training	Can you think of any barriers preventing Community Health Workers or Promotoras from participating in the training (online or in-person)? Why?
	What would be the most likely factors to facilitate your participation in the training when it does become available (online or in-person)? Why?
Recognitions	Do you think it is important to receive some certification for completing the training or “badges” that can be posted on linkedin or on a resume, to show that you have completed training in some or all of the modules?
Peer-led training	Do you like the idea of peer-led training? Why? Why not?

### Data Analysis

The transcribed data were analyzed using the Rigorous and Accelerated Data Reduction (RADaR) technique [18]. The RADaR technique involves using spreadsheets to develop all-inclusive data tables that undergo several revisions called “data reduction,” which yield shorter, more concise data tables. A team of three data analysts led by the first author (DV) worked on reducing transcribed data into data tables. Codes were assigned to the final data table based on the research questions proposed and discussed during the weekly meetings among the analysts.

New codes were generated whenever any of the participants presented new information; these codes were discussed during the weekly coding meeting and finalized. During the coding cycles, we identified and compiled similar codes under themes. For example, several unique codes regarding specific additions to training content were all assigned to one code for, “recommended additions to training content.” The results from the qualitative data are presented below based on analyses of final themes and corresponding subthemes.

## Results

Demographic characteristics of the CHWs/Ps are shown in Table 2. Forty CHW/Ps (38 women, 2 men, mean age 43.5) participated. More than half of the sample identified as Hispanic/Latino ethnicity (58%). Regarding race, half of the sample identified as White (50%), followed by 28% who identified as Black and 15% who identified as Other (which included Hispanic/Latino individuals who did not identify with the racial categories provided). There was a broad range of years of CHW/P work experience with 32.5% of the sample for more than 10 years and only 10% working for less than one year.

**Table 2.** Sample characteristics (N = 40)

Characteristic	n	%
Race		
White Black Other Multi-race Native American	20 11 6 2 1	50 27.5 15 5 2.5
Ethnicity		
Hispanic	23	57.5
Sex		
Female	38	95
Age		
20s 30s 40s 50s 60s 70s Unknown	8 6 12 8 4 1 1	20 15 30 20 10 2.5 2.5
Years Working as a Community Health Worker or Promotora		
Less than 1 year 1-3 years 3-5 years 5-10 years More than 10 years Unknown	4 9 7 6 13 1	10 22.5 17.5 15 32.5 2.5

Analyses of focus group data led to the validation of each of the four themes that the study team agreed upon based on its research objectives and the identification of corresponding subthemes. These results are presented for each of the four themes as follows: overall relevance of the training, benefits of certification, preference for flexible delivery of the training, and peer-led training: barriers and facilitators. The distinct subthemes associated with each theme are also shown in Table 3.

**Table 3.** List of themes, codes, and their definitions

List	Theme	Codes	Definitions
1	Relevance of training	Periodic training needed	Participants mentioned the need for periodic training
		Provide tools and skills	Participants mentioned training provides them with the tools and skills to engage with the community meaningfully
		Relevant for all	Participants mentioned the relevance of training for new and experienced CHW/Ps
		Increase knowledge	Participants mentioned training helps increase knowledge
2	Benefits of training completion certificates	Personal and professional benefits	Participants discuss the benefits of receiving a course completion certificate on both personal and professional levels
3	Preferred delivery methods of training modules	Both needed	Participants mentioned in-person and online training is needed
		Advantages of online training	Participants explain the advantages of online training
		Disadvantages of online training	Participants explain the disadvantages of online training
		Advantages of in-person training	Participants explain the advantages of in-person training
		Disadvantages of in-person training	Participants explain the disadvantages of in-person training
4	Peer-led training	Facilitators	Participants discuss the relevance of peer-led training
		Barriers	Participants discuss barriers to peer-led training

Note. CHW/P = Community Health Worker/Promotora.

### Relevance of CHW/P Training Modules

Most focus group participants confirmed that such culturally and linguistically appropriate health research training would be relevant to their work. These are some quotes from focus group participants:

*[CHW/P training] is really important because we don't always come from the same backgrounds…we are not always doing the same type of work and we have not been doing this for a really long time…it is beneficial not just for Community Health Workers, but also for the employers. This [training] is going to directly impact your results. (36, F, Hispanic/Latino, White, more than 10 years CHW/P)*

*It is good to have it [CHW/P training] under your belt to say I know how to do that; it makes you feel a little bit more comfortable. You can go back to these tools and use them throughout your careers as a Community Health Worker with whatever study it might be. (44, F, Not Hispanic/Latino, Black, 1-3 years CHW/P)*

Many participants also mentioned needing the training to equip them with tools and skills they used at work. These skills were described as being necessary to foster effective bidirectional communication with diverse communities. However, some participants also noted a need for such training that was appropriate for different cultures and languages they encountered.

*We need training to know what the content is, [to] give us tools. Another challenge is that people sometimes do not have the same level of education…they cannot read…they cannot write. They do not speak English…they do not speak Spanish either. I have met people who speak Mayan. (33, F, Hispanic/Latino, Other, 3-5 years CHW/P)*

*I also feel that it will be useful for the Community Health Workers because they’re going to educate themselves to educate people. (46, F, Hispanic/Latino, White, 10 years CHW/P)*

A few participants also described the research training as being appropriate for both new and experienced CHW/Ps.

*When we are new to the profession, sometimes we do not always know everything that there is to know about research programs. [For those of us] who have been doing it for a while, it’s just a good reminder to remember why it is that we need to keep the data clean and the purpose of these research programs. (36, F, Hispanic/Latino, White, more than 10 years CHW/P)*

Importantly, some participants linked CHW/P training with their own opportunity to build more trusting relationships with research participants in the communities they served. And others noted that the training could enable them to better explain research studies with confidence.

*I reinforce what I said from the beginning, the importance of us being trained and including us as Community Health Workers at the beginning of the research. Because when we are convinced of what we are offering to our participants, we achieve that connection, trust, and openness to them. For us to be able to give that information to them, we want to be very prepared, very educated to go out as they say--go to fight for them. (47, F, Hispanic/Latino, Other, 3-5 years CHW/P)*

*They [CHW/Ps] could recruit more people by giving confidence by explaining all the sections and by being clear. If the community trusts them, they let other people know, and recruitment and participation is better… I think the workers would be more confident with this training about how they are working. (46, F, Hispanic/Latino, White, 10 years CHW/P)*

*It opens the channels and you will have more confidence with the person you are recruiting, more open. (54, F, Hispanic/Latino, 5-10 years CHW/P)*

### Benefits of Training Certificates

Notably, a small proportion of the participants strongly emphasized that those who completed the CHW/P training could benefit from being provided with a certificate that states they have received the training. Some participants described the benefit of such certification in personal terms by characterizing it as valued recognition.

*Well, pretty much any moment that you can share your accomplishments and be able to help the next person and also make you as well as the organization that you work for valuable. It would be beneficial for the next person, including yourself.*_*…*_ *Because we have a heart for it, you know we don't do it necessarily to get a congratulations and a pat on our back, but that’s always appreciative as well. Yeah [it] keeps us going and everything else. (49, F, Not Hispanic/Latino, Black, 3-5 years CHW/P))*

*An acknowledgment always feels good. (46, F, Hispanic/Latino, White, 10 years CHW/P)*

In contrast, a few other participants emphasized that providing certificates of completion could yield professional benefits for the participants, including but not limited to their continuing education and career advancement.

*I am not sure about the rest of the Community Health Workers, but congratulations or awards are not that important to us. We just need the certification so we can just do our jobs better. (41, F, Not Hispanic/Latino, Black, 3-5 years CHW/P)*

*It just adds to your portfolio…Just as when you go to the next step that this is what you have done, and this is what you bring to the current organization and then in the future. Therefore, it is always good to have the accolade or piece of paper saying that you have completed something…You get more buy in when you have a trained trainer. (58, F, Not Hispanic/Latino, Black, more than 10 years CHW/P)*

### Preferred Delivery Methods of CHW/P Training Modules

The ways in which the CHW/P training would be delivered was discussed at length in all six focus groups and addressed by the vast majority of the participants. Several participants mentioned that the availability of both online and in-person training would be desirable by CHW/Ps since it provides them with different options from which they could choose to fit their circumstances. One participant emphasized that having both online and in-person training would allow CHW/Ps to choose and take the training at their own pace as well.

*Not everyone learns the same, some people prefer to do it in person, and they can do it online on their own and faster. I like that they offer that option. Sometimes it’s more convenient to do it online rather than in person, but some people prefer to review it more times and it helps them better grasp or learn the information. (46, F, Hispanic/Latino, White, 10 years CHW/P) I think that it’s a great opportunity because we don't all learn the same way, for those who need in-person or more hands-on, I think the instructor-led would be great, and I don't think I need hands-on training for each topic, but maybe there are specific ones that I think I would do better in person. I think it’s great that you're offering the opportunity to do both if needed. (36, F, Hispanic/Latino, White, more than 10 years CHW/P)*

Although in-person training options were highlighted as being preferred in some circumstances, several participants specifically highlighted the advantages of an online training module for busy CHW/Ps who may not be able to attend in-person training.

*I imagine you can do it at your own time if it’s online. I don’t think there’s any impediment, unless it’s something technical, if you don’t have internet, if you don’t have a computer, but I don’t think there’s anything else outside of that.* (54, F, Hispanic/Latino, Other, 5-10 years CHW/P)

*From my point of view, for me it would be more practical virtually, and that’s one of the great advantages that the pandemic has brought for me. Because we can make it virtual, more people have been able to connect at the same time from the comfort of their home because we know that at this time there are many barriers, such as transportation, time, children. Virtual for me is fine.* (44, F, Hispanic/Latino, White, 3-5 years CHW/P)

However, the lack of access to internet connectivity and computer technology among some of the community members served by CHW/Ps was also mentioned as one of the disadvantages associated with the exclusive use of online training modalities.

*When we talk about rural communities, they do not always have access, and we have to consider that. There are people who still have a lot of problems with the access to the internet and who are still learning all this from Zoom. They have problems and if they have a question, who are they going to turn to? Even if it was virtual, if that’s the decision, they need to have a point of contact, someone who supports them both in the technical and in questions that may arise about the training. (32, F, Hispanic/Latino, White, 3-5 years CHW/P)*

Some participants did note the advantages of instructor-led trainings over virtual training in particular circumstances, although many emphasized that instructor- and in-person trainings may require a greater commitment of resources and time.

*I think it would be more enriching to have an instructor in case we have any questions. Even if the topic is very well-explained, and mainly that it’s a new topic, we’re always going to have questions. When there’s a prepared instructor who can dispel the doubt, and in case she can’t, take that question and she’d see who else to lean on, but this person wouldn’t be left with the doubt. This would be good for me (44, F, Hispanic/Latino, White, 3-5 years CHW/P)*

*In person it’s a bit more difficult because you have to combine it with the other participants and you have to attend [at] the place and have time; it’s less flexible. (54 years, F, Hispanic/Latino, Other, 5-10 years CHW/P)*

*I think supervisors are always open to [in-person training], but I would think that the cost to have in person training is pretty expensive and most supervisors, at least my supervisors, probably would prefer an online portion that I could just get in between clients or on my own time. (39, F, Not Hispanic/Latino, White, 5-10 years CHW/P)*

*You may want to consider doing the instructor-led, maybe two days. Like a part one and part two, for those who may not have that three-hour block available…because a lot of times people don't have three hours for instructor led to commit to. (58, F, Not Hispanic/Latino, Black, more than 10 years CHW/P)*

### Peer-led trainings: Facilitators & Barriers

Most participants liked the idea of peer-led training. The main advantages reported were the ability to share and socialize within a small group, to hear directly from people who are working in the field, to increase "buy-in" from the trainees, and to improve support.

*(I like it) because the group is small, but you have someone else to share or socialize with. (46 years, F, Hispanic/Latino, White, 10 years CHW/P)*

*You get more buy-in when you have a trained trainer. And it’s fun to have two people, two different styles, it’s just a great way of delivering training, I think. (58, F, Not Hispanic/Latino, Black, more than 10 years CHW/P)*

*You support each other to share your doubts. If you have a meeting and you forget, someone reminds you. (54 years, F, Hispanic/Latino, 5-10 years CHW/P)*

A few additional participants mentioned the importance of hearing from those who have actual lived experience conducting community outreach in local communities.

*I think a (peer) trainer is always good…because you are actually getting to hear from the horse’s mouth. You're getting [to hear from] the foot soldiers, who are doing the work on the ground, and who have the experience that we share as Community health Workers… (73, M, Not Hispanic/Latino, Black, more than 10 years CHW/P)*

*I do feel really strongly about the peer led training. Simply because of the whole idea of nothing about us without us and so on… You know just always keeping us in the center of everything because as community health workers, clinical health researchers, and promotoras, we need to understand how to do the research, because it is directly affecting us. And there really isn't a good reason that we couldn't have peer-led. Someone who is right there with you….(because) we always joke about that person sitting up in the high tower just coming in telling you how to do it and what to do. (51, F, Not Hispanic/Latina, White, more than 10 years CHW/P)*

One participant highlighted that peer-led trainings would be preferred, especially by people who are from the same community or by people who have gone through similar experiences historically. These peer trainers would have a clearer understanding of the purpose of research and improve participation.

*I was thinking that the peer-led [training] is good because of what we are going through right now. Research is important. Just think about the African Americans dealing with the vaccinations right now with the COVID 19, and what we went through. Some even heard about Tuskegee, the research that they did on the men with syphilis. I think it is affecting our mindset at this point whether we should get [the vaccine]. In the African American community, vaccination is really low at this time, and it has always been like that, because of this… I think this peer leading training would be really so good to help folks understand more about research and what it does, on the other end. (58, F, Not Hispanic/Latino, Black, more than 10 years CHW/P)*

Some participants pointed out that a lack of adequate knowledge or the ability to communicate well could be a barrier to benefiting from peer-led training. However, others suggested information dissemination by a person whom they trust, such as through a peer-led training, is better received than from a doctor or from media.

*If the peers that are providing the education, don't have enough knowledge then they might not be able to answer questions, or maybe they might not be able to express well…(36, F, Hispanic/Latino, White, more than 10 years CHW/P)*

*I think that there are pros and cons to peer led training. We did a peer led training with our participants about COVID Vaccine and that went off very well. It was received a little better than hearing it on the news or hearing doctors talk about COVID or hearing from someone on the news you don't trust or who you don't think have had an understanding or your wellbeing at heart, sometimes can be hard… (44, F, Not Hispanic/Latino, Black, 1-3 years CHW/P)*

Another participant mentioned that though she likes peer-led training, sometimes she prefers training by experts who could answer her questions and clarify common myths.

*At the same time [when it is a peer-led training] you can still feel that someone who is not a professional or that is not their subject matter expert, that they might not have the actual details. Sometimes I do like subject matter experts to tell me what’s going on and to have the details behind it and to be able to answer those myths and questions and things that might occur. (44, F, Not Hispanic/Latino, Black, 1-3 years CHW/P)*

## Discussion

We designed and implemented a focus group study in partnership with CHW/Ps to gain a deeper understanding of how best to ensure the effective dissemination of culturally and linguistically appropriate health research training. The results can facilitate the creation of a research best practices training for this workforce that will be relevant, beneficial, and feasible to participate in. CHW/Ps overall felt this type of training was important to their work engaging the communities they serve in health research.

It may be that CHW/Ps are seeing an uptick in research opportunities within the communities they serve, particularly with regard to research on the effects of the COVID-19 pandemic on diverse communities. The pandemic has exacerbated health inequities in underserved communities served by CHW/Ps [19,20]. While federal agencies are prioritizing efforts to reduce the spread and health effects of COVID-19, CHW/Ps are increasingly being recognized for their crucial role in assisting with health promotion and research efforts with the pandemic in underserved communities [21–23].

In general, CHW/Ps are increasingly recognized for their ability to help build and maintain trust with people that have historically mistrusted the health care system [22]. These dynamics will only accelerate the ongoing shift in the CHW/P role from being health educators to being health care delivery workers and health research study recruiters [24]. This could be another reason for CHW/Ps in this study stating that periodic refresher training is indeed useful to keep themselves up to date on research opportunities and developments that could be conveyed effectively to the communities that they serve.

The myriad roles played by CHW/Ps require training that serves not only to improve their professional knowledge and skills but also can increase their confidence to engage effectively with the communities they serve and to improve communication skills in discussing topics related to health research. Many CHW/Ps may experience “task shifting” as per the evolving needs of the communities they serve. This may be particularly likely to occur during public health crises such as the COVID-19 pandemic, in which duties of CHW/Ps may range from educating and delivering care safely by participating in vaccination drives, to acting as trusted sources of information for communities during times of uncertainty that necessitate rapid and rigorous research.

CHW/Ps who work with minoritized groups and historically underserved populations have a job that can be particularly challenging [22]. As mentioned by our participants, culturally and linguistically appropriate training that helps to build capacity to perform their job tasks is important, relevant, and critical in such situations. Becoming knowledgeable about the community’s culture, economic conditions, social networks, political and power structures, norms and values, demographic trends, history, and experience with efforts by outside groups are an important principle of community engagement [4]. In the current study, the results support the notion that attention to cultural and linguistic appropriateness of training for CHW/Ps can help ensure its relevance, especially for training designed to be disseminated to CHW/P’s who serve diverse community members for the purpose of advancing health research and bridging health inequities.

The ability to provide culturally and linguistically appropriate training for CHW/Ps is necessary as the US population becomes more ethnically and racially diverse over the next 40 years [25]. With the shifting demographic trends of the United States, CHW/Ps will benefit from this tailored training to optimally support community-engaged research. As recruitment and retention of participants is often a main function of CHW/Ps who work on research teams [7], the delivery of culturally and linguistically appropriate training may promote understanding and professional practices that ultimately serve to increase the rigor and efficacy and effectiveness of health research studies conducted with diverse communities across the country.

### Delivery Format

The participants mentioned that a flexible modality of CHW/P training is beneficial, especially if it involves training being offered in a hybrid format- both in-person and online. Such flexibility may provide an opportunity for a CHW/P to choose what works for them based on their schedule. This reinforces one of the most important principles of community engagement which highlights the need for organizations that wish to engage with the community to be flexible enough to meet its changing needs [4]. For example, with the COVID-19 pandemic, online trainings and other virtual meetings have become commonplace, which has resulted in increased access and comfort with online trainings among many in the clinical and translational research workforce. A recently completed study on training needs of CHWs during pandemic also reported the preference for short, self-paced, online courses, especially for continuing education units (CEUs), needed to maintain certification by CHW/Ps [22].

Online trainings that allow individuals to complete training per their convenience may be particularly important to promoting CHW/P professional development. In fact, WHO guidelines on CHW/P training recommends that online learning can supplement other training modalities and is particularly appropriate for follow-up and refresher training [26]. Online training has been found to be cost effective and an effective method to improve exchange of knowledge between CHW/Ps who work at different geographic locations [27]. Indeed, empirical research has shown substantial cost savings of over 40% between baseline and blended training programs for training CHWs [28]. In addition, use of multimedia materials, visuals, and audio has been shown to be helpful for the CHW/P workforce which consists of diverse individuals with different educational background and experience [28].

The flexibility afforded to CHW/Ps by an online learning format was emphasized by several participants as being an advantage over in-person format. They further noted that lack of internet access, unfriendly user interface, absence of assistance with technological issues, and one’s own limited technological experience could be substantive barriers to successful online learning. However, many also expressed their preference for peer-led trainings where an experienced co-worker or a peer explains difficult concepts to them in-person with examples or allows them to shadow during outreach to provide a firsthand experience to the new CHW/P.

Previous studies have shown that peer-led participatory training models encourage participants to more freely share their opinions, provide opportunities for open dialogue and for a guided review of information, and make informed decisions [29,30]. It may also make training more enjoyable if CHW/Ps share personal experiences and learn to apply principles learned from everyday life. Having a hybrid format where online learning is augmented with periodic in-person sessions could provide the CHW/Ps with hands-on experience as well as offset some of the technology-related barriers of a purely online format.

### Benefits of Certificates

A few participants expressed a preference for having proof of training completion and a few others noted that earning such recognition could help them advance in their career. Also it is reasonable to believe that having evidence of completing this training may be viewed positively by employers or a way to broadly demonstrate their proficiency in crucial area of expertise. For example, such evidence of completing the training could be used by CHW/Ps to show their familiarity with research best practice principles and thereby increase their ability and confidence to partner with researchers working in the communities they serve. There are mixed opinions about providing "certifications" to CHW/Ps followed by formal training [6,32].

Based on findings from this study, providing participants with the option of receiving a certificate of completion may help them demonstrate their proficiency as CHW/Ps or otherwise help them to demonstrate their knowledge and skillsets. This could help increase their self-confidence while interacting with community members and research teams. Importantly, providing such certificates might serve to further empower CHW/Ps ability to transfer their knowledge to other CHW/Ps, an act which is often needed for effective community-engaged research.

Professionalizing the CHW/P role requires recognition of roles and competencies and to be trained accordingly. While there is not a recognized national set of training standards for CHW/Ps, efforts to define roles and the necessary skills to fulfill those roles are underway across many states and organizations [32]. The training described here provides an important means to educate CHW/Ps and can provide the curricular content from which to develop measurable assessments of research skills for the CHW/P workforce.

## Strengths & Limitations

We believe that this study fills a gap in the literature regarding the relevance and optimal dissemination of CHW/Ps research best practices training in historically underserved and minoritized communities across the entire USA. Indeed, the parent project of this focus group study aims to develop and broadly disseminate culturally and linguistically-appropriate research best practices training for CHW/Ps. A strength of this study is the participatory approach utilized to solicit and better understand the perspectives of English and Spanish-speaking CHW/Ps that is being used to guide dissemination of the best training practices course modules. This study is potentially limited in generalizability given that we are only testing the course in a few study sites. Additionally, the focus was on English- and Spanish-Speaking CHW/Ps in this project, so study findings can only be partially generalized to these groups.

## Conclusion

Our findings indicate that the CHW/Ps perceive that their research role necessitates a periodic, standard training with flexible delivery and peer-led instruction and available mentoring. The attention to cultural and linguistic appropriateness of the training is designed to increase uptake, use, and utility of the course.

CHW/Ps are quickly becoming an instrumental and well-recognized component of the clinical and health research workforce [1–3]. There is a parallel and growing demand for quality training for CHW/Ps which is culturally- and linguistically appropriate to those aspects of their work which contribute to health research. It is essential that new research training opportunities are developed for these professionals which further enables them to understand and represent the health and research priorities of the diverse communities in which they live and work.
